# Contribution of Zinc-Dependent Delayed Calcium Influx via TRPC5 in Oxidative Neuronal Death and its Prevention by Novel TRPC Antagonist

**DOI:** 10.1007/s12035-018-1258-7

**Published:** 2018-07-31

**Authors:** Sang Eun Park, Ji Hoon Song, Chansik Hong, Dong Eun Kim, Jee-Won Sul, Tae-Youn Kim, Bo-Ra Seo, Insuk So, Sang-Yeob Kim, Dong-Jun Bae, Mi-Ha Park, Hye Min Lim, In-Jeoung Baek, Antonio Riccio, Joo-Yong Lee, Woo Hyun Shim, Bumwoo Park, Jae-Young Koh, Jung Jin Hwang

**Affiliations:** 1 Asan Institute for Life Sciences, Asan Medical Center, Seoul, 05505 South Korea; 2 Department of Physiology, Chosun University School of Medicine, Kwangju, 61452 South Korea; 3 Neural Injury Research Lab, University of Ulsan College of Medicine, Seoul, 05505 South Korea; 4 Department of Physiology and Institute of Dermatological Science, Seoul National University College of Medicine, Seoul, 110-799 South Korea; 5 Department of Convergence Medicine, University of Ulsan College of Medicine, 88, Olympic-ro 43-gil, Songpa-Gu, Seoul, 05505 South Korea; 6 Department of Cardiology, Boston Children’s Hospital, Boston, MA USA; 7 Department of Neurobiology, Harvard Medical School, Boston, MA USA; 8 Biomedical Engineering, Ulsan National Institute of Science and Technology, Ulsan, 44919 South Korea; 9 Department of Neurology, Asan Medical Center, University of Ulsan College of Medicine, 88, Olympic-ro 43-gil, Songpa-Gu, Seoul, 05505 South Korea

**Keywords:** Ca^2+^, H_2_O_2_, NU6027, Seizure, TRPC, Zn^2+^

## Abstract

Oxidative stress is a key mediator of neuronal death in acute brain injuries, such as epilepsy, trauma, and stroke. Although it is accompanied by diverse cellular changes, increases in levels of intracellular zinc ion (Zn^2+^) and calcium ion (Ca^2+^) may play a critical causative role in oxidative neuronal death. However, the mechanistic link between Zn^2+^ and Ca^2+^ dyshomeostasis in neurons during oxidative stress is not well-understood. Here, we show that the exposure of cortical neurons to H_2_O_2_ led to a zinc-triggered calcium influx, which resulted in neuronal death. The cyclin-dependent kinase inhibitor, NU6027, inhibited H_2_O_2_-induced Ca^2+^ increases and subsequent cell death in cortical neurons, without affecting the early increase in Zn^2+^. Therefore, we attempted to identify the zinc-regulated Ca^2+^ pathway that was inhibited by NU6027. The expression profile in cortical neurons identified transient receptor potential cation channel 5 (TRPC5) as a candidate that is known to involve in the generation of epileptiform burst firing and epileptic neuronal death (Phelan KD et al. 2012a; Phelan KD et al. 2013b). NU6027 inhibited basal and zinc-augmented TRPC5 currents in TRPC5-overexpressing HEK293 cells. Consistently, cortical neurons from TRPC5 knockout mice were highly resistant to H_2_O_2_-induced death. Moreover, NU6027 is neuroprotective in kainate-treated epileptic rats. Our results demonstrate that TRPC5 is a novel therapeutic target against oxidative neuronal injury in prolonged seizures and that NU6027 is a potent inhibitor of TRPC5.

**Electronic supplementary material:**

The online version of this article (10.1007/s12035-018-1258-7) contains supplementary material, which is available to authorized users.

## Introduction

Reactive oxygen species (ROS) play important pathological roles in numerous neurological disorders, such as seizure, ischemic stroke, and brain and spinal cord trauma [1–3]. Because the brain consumes a considerable amount of oxygen and contains a high concentration of polyunsaturated fatty acid that is easily oxidized, it is particularly susceptible to oxidative stress [4]. During oxidative stress, the concentration of intracellular calcium ions ([Ca^2+^]_*i*_) gradually increases, leading to neuronal death. A variety of Ca^2+^ channels are involved in the elevation of [Ca^2+^]_*i*_ during neuronal injuries, including ionic glutamate receptors [*N*-methyl-d-aspartate (NMDA), alpha-amino-3-hydroxy-5-methyl-4-isoxazolepropionic acid (AMPA), and kainic acid (KA) receptors], metabotropic glutamate receptors, voltage-dependent calcium channels, and the transient receptor potential (TRP) channel family in plasma membrane as well as inositol trisphosphate (IP3) receptors and ryanodine receptors in endoplasmic reticulum [5]. Although the overactivation of glutamate receptors induces increases in [Ca^2+^]_*i*_ during neuronal death, TRP channels may also mediate oxidative stress-induced increases in [Ca^2+^]_*i*_ in case of brain injuries [6].

Mammalian TRP channels belong to a family of Ca^2+^-permeable nonselective cationic channels [7]. TRP channels are further grouped into seven subfamilies: TRPC, TRPM, TRPV, TRPA, TRPP, TRPML, and TRPN. These TRP channels have been implicated in many physiological events, including development and neuroplasticity [8]. TRP channels also play important roles in neuronal death, such as capsaicin-triggered TRPV1 activation in mesencephalic dopaminergic neuronal death [9], amyloid β- and H_2_O_2_-induced TRPM2 activation in striatal cell death [10], and ROS-mediated TRPM7 activation in ischemic neuronal injury [11]. Recently, there has been an increasing interest in TRPC1/4/5 in epileptogenesis and neuronal death. TRPC channels comprise seven isotypes (TRPC1–TRPC7). Of these, TRPC4 and TRPC5 are abundantly expressed in the brain [12] and may be involved in epileptiform burst firing and epileptic neuronal death [13, 14]. In addition, S-glutathionylation of TRPC5 and downregulation of TRPC1 during oxidative stress may be involved in neuronal death in Huntington’s disease [15]. Despite some scattered evidence, the role of TRPCs in neuronal death has not drawn much attention.

Zinc ions (Zn^2+^) also play physiological and pathological roles in the central nervous system. Under physiological conditions, intracellular Zn^2+^ is tightly regulated by zinc transporters (ZnTs), ZRT, IRT-like proteins, and buffering proteins, such as metallothioneins [16]. However, excessively high levels of intracellular free Zn^2+^ in seizures, stroke, or trauma trigger neuronal death [17]. Interestingly, the two potentially toxic events, Ca^2+^ and Zn^2+^ dyshomeostasis, are correlated. Several papers have suggested that increases in intracellular Zn^2+^ contribute to the subsequent increase in Ca^2+^ [18, 19]. Moreover, increasing intracellular Zn^2+^ by clioquinol and pyrithione, Zn^2+^-ionophores can activate TRPA1 channels [20]. Hence, initial Zn^2+^ dyshomeostasis may trigger Ca^2+^ dyshomeostasis, which together causes neuronal death under injuries.

Although ample evidence supports that oxidative stress is a key mechanism contributing to neuronal death in acute brain injury, a variety of clinical trials with drugs targeting ROS have been unsuccessful [21]. For instance, potent antioxidants, such as *N*-acetyl cysteine and NXY-059, were not beneficial in patients with epilepsy or ischemic stroke [22, 23]. There are many possible reasons for these failures, including weak antioxidant capacity, poor blood–brain barrier penetration, and rapid clearance in vivo [24]. Despite these failures, ROS are major targets for neuroprotective drugs, and new insights into the toxic mechanisms of oxidative injury are required.

While searching for neuroprotective drugs effective against oxidative stress-induced cell death, we found that NU6027 showed marked protective effects. NU6027 blocks neuronal death induced by H_2_O_2_ and KA in primary cortical cultures and in a rat seizure model, respectively, via a novel mechanism, the inhibition of TRPC5.

## Materials and Methods

### Primary Mouse Cortical Cell Cultures

Pure astrocyte cultures were prepared from postnatal day 3 (P3) ICR mice and maintained in Dulbecco’s modified Eagle’s medium (Gibco) supplemented with 5% fetal bovine serum (Hyclone), 5% horse serum (Gibco), 2 mM glutamine (Sigma), and 1% penicillin/streptomycin (Cambrex). These astrocyte cultures were used for experiments or as feeder cells for mixed cortical cultures. Mixed cortical cultures were prepared by plating cortical neurons from embryonic day 14 (E14) ICR mice onto pure astrocyte cultures and growing them in growth media. Pure neuronal cultures were prepared from cortices of E14 ICR mice or age-matched wild-type (WT) or TRPC5 knock-out (KO) 129/SvImJ mice and were grown in neurobasal media (Gibco) containing B27 supplement (Gibco), 2 mM glutamine, and antibiotics.

### Exposure to H_2_O_2_ and Other Reagents

Cells were exposed to glutamate, H_2_O_2_ (Sigma), sodium nitroprusside (SNP, a donor of nitric oxide, Sigma), and ZnCl_2_ (Sigma) in minimum essential media (Gibco) for 24 h at the indicated concentrations to induce cell death. Anthranilic acid, clotrimazole, dantrolene, flufenamic acid, kenpaullone, NU6027, olomoucine, roscovitine, ruthenium red, SU9516, *N*,*N*,*N*,*N*′-tetrakis (2-pyridylmethyl) ethylenediamine (TPEN), and 2-aminoethyl diphenylborinate (2-APB) were purchased from Sigma. Capsaicin, MK-801, ML204, Pyr3, and (−)-Xestospongin C (XeC) were purchased from Tocris. SB216763 was purchased from Enzo Life Science, and 6-cyano-7-nitroquinoxaline-2,3-dione (CNQX) was purchased from RBI. All reagents were added 1 h prior to H_2_O_2_ exposure, unless otherwise stated.

### Assessment of Cell Death

Lactate dehydrogenase (LDH) released into culture media from damaged cells was measured to evaluate cell death [25]. The mean background value in control sister cultures that only received a sham wash (0% cell death) was subtracted from the LDH value in each test condition, and the LDH value was scaled to the mean value of sister cultures after 24 h of exposure to 200 μM glutamate, which resulted in nearly complete neuronal death without astrocytic damage (100%). Cell death was also detected by staining with 2 μg/ml propidium iodide (PI, Sigma) at 37 °C in a CO_2_ incubator for 10 min. Images were obtained using a fluorescent microscope (Olympus, IX71) equipped with a CCD camera (Olympus, IX-10) at a wavelength of 535/617 nm (ex/em).

### Live Cell Imaging for Zn^2+^ and Ca^2+^

To label Zn^2+^, pure neuronal cultures were treated with 2.5 μM FluoZin-3 AM (Invitrogen) for 30 min before imaging. To detect Ca^2+^, cultures were transfected 2 days before experiments with 10 μg of pCMV-RGECO1 plasmid (R-GECO1, a genetically encoded Ca^2+^ indicator) using Lipofectamine 2000 (Invitrogen). Live cell images were obtained using an inverted fluorescent microscope (Ti-E; Nikon) equipped with a Cascade 212B camera (Roper Scientific), and images were acquired every 1 min at wavelengths of 494/516 nm and 570/595 nm (ex/em) for FluoZin-3 AM and R-GECO1, respectively. Images of Ca^2+^ staining were also obtained by staining cells with 2 μM Fluo-4 AM (Invitrogen) for 30 min. The images were obtained with an inverted fluorescent microscope (Olympus, IX71) equipped with a CCD camera (Olympus, IX-10) at a wavelength of 494/506 nm (ex/em). Fluorescence intensity was analyzed by Image J software and represented as the fold increase compared with control.

### Reverse Transcription-Polymerase Chain Reaction (RT-PCR)

Total cellular RNA was extracted from cultured pure neurons and astrocytes using Trizol (Invitrogen) according to the manufacturer’s instructions. Complementary DNA (cDNA) was synthesized using the RT^2^ First Strand kit (Qiagen). PCR reactions were prepared by mixing equal amounts of cDNA, a set of primers for mouse TRPC subtypes, and actin (Table 1) into the AccuPower ProFi Taq PCR premix (BioNeer). PCR was carried out in a C1000 Thermal Cycler (Bio-Rad) for 30 cycles (denaturation at 95 °C for 20 s, annealing at 55 °C for 20 s, and extension at 72 °C for 20 s). PCR products were separated on 1.5% agarose gels and visualized on a Geldoc (Bio-Rad).

**Table 1:** List of primers used for RT-PCR

Genes	Forward primer (5′ → 3′)	Reverse primer (5′ → 3′)	Accession number
TRPC1	TGGGATGATTTGGTCAGACA′	CCAATGAACGAGTGGAAGGT	NM_011643
TRPC2	AGCCTCAGTACATTGCCCTG	AAGTTCCACCAGTCCAGGAG	NM_011644
TRPC3	AGAGCGATCTGAGCGAAGTC	TTTGGAACGAGCAAACTTCC	NM_019510
TRPC4	ACGCGTTTTCCACGTTATTC	CTTCGGTTTTTGCCTCTCTG	NM_016984
TRPC5	ATTATTCCCAGCCCCAAATC	GACAGGCCTTTTTCTTGCAG	NN_009428
TRPC6	AAAGATATCTTCAAATTCATGGTC	CACGTCCGCATCATCCTCAATTTC	NM_013838
TRPC7	CGTGCTGTATGGGGTTTATAATG	GCTTTGGAATGCTGTTAGAC	NM_012035
Actin	TGTTACCAACTGGGACGACA	AAGGAAGGCTGGAAAAGAGC	NM_007393

### Immunofluorescence

Cultured pure neurons and astrocytes were fixed with 4% paraformaldehyde for 15 min, permeabilized with 0.1% Triton X-100 for 5 min, and blocked with 1% bovine serum albumin for 30 min. For staining, cells were incubated with antibodies for glial fibrillary acidic protein (Millipore, AB5807), MAP2 (Abcam, AB32454), and TRPC5 (Neuromab, 75-104) at 4 °C overnight, followed by incubation with fluorescence-labeled secondary antibodies for 2 h. For nuclear staining, cells were incubated with 5 μg/ml Hoechst 33342 (Sigma). Cells were mounted and visualized under the EVOS Cell Imaging System (Thermo Fisher Scientific).

### Immunoblot Analysis

Membrane proteins from cultured pure neurons and astrocytes were extracted with lysis buffer (20 mM Tris-HCl pH 7.4, 150 mM NaCl, 1 mM EDTA, 0.5% SDS, 2.5 mM sodium pyrophosphate, 1 μM NaVO4, and protease inhibitors). Proteins were separated by 8% SDS-polyacrylamide gel electrophoresis and transferred to polyvinylidene difluoride membranes (Millipore). Membranes were probed with antibodies to TRPC5 (Neuromab, 75-104) and β-actin (Sigma, A0560), followed by incubation with the appropriate secondary antibody conjugated to horseradish peroxidase (Thermo Fisher Scientific). Immunoreactivity was visualized using Immunobilon™ Western Chemiluminescent HRP Substrate (Millipore) and the Kodak Image Station 4000MM (Kodak).

### Electrophysiological Analysis

For TRPC5 current recordings, human embryonic kidney (HEK) 293 cells (ATCC) were maintained according to the manufacturer’s recommendations and transfected with plasmid DNA expressing mouse TPRC5 (pIRES-mTRPC5-GFP) using FuGENE6 (Roche). Whole-cell currents were recorded using an Axopatch 200B amplifier (Axon Instruments). Currents were filtered at 5 kHz (3 dB, 4-pole Bessel), digitized using a Digidata 1440A Interface (Axon Instruments), and analyzed using a personal computer equipped with pClamp 10.2 software (Axon Instruments) and Origin software (Microcal Origin v. 8.0). Patch pipettes were made from borosilicate glass and had resistances of 2–4 MΩ when filled with standard intracellular solutions. We used an external bath medium (normal Tyrode solution) of the following composition: 135 mM NaCl, 5 mM KCl, 2 mM CaCl_2_, 1 mM MgCl_2_, 10 mM glucose, and 10 mM *N*-[2-hydroxyethyl]piperazine-*N*′-[2-ethanesulfonic acid] (HEPES), with pH adjusted to 7.4 using NaOH. A Cs^+^-rich external solution was made by replacing NaCl and KCl with equimolar CsCl. The standard pipette solution contained 140 mM CsCl, 10 mM HEPES, 0.2 mM Tris-GTP, 0.5 mM EGTA, and 3 mM Mg-ATP, with the pH adjusted to 7.3 using CsOH. After TRPC5 activation in a Cs^+^-rich solution, 10 μM NU6027 was externally applied at the time indicated by the bars. ZnCl_2_ was intracellularly applied via the pipette solution. Voltage ramp pulses were applied from + 100 to − 100 mV for 500 ms at a holding potential of − 60 mV. The junction potential between the pipette and bath solutions used for all cells during sealing was calculated to be 5 mV (pipette negative) using pClamp 10.2 software. No junction potential correction was applied. Experiments were performed at room temperature (18 °C–22 °C). Cells were continuously perfused at a rate of 0.5 ml/min. The inward current amplitudes of all bar graphs and current traces were taken during the ramp pulses at a holding potential of − 60 mV.

#### Breeding and Genotyping of TRPC5 KO Mice and Pure Neuronal Cultures

TRPC5 KO mice in our experiments were provided by Dr. Antonio Riccio at the Howard Hughes Medical Institute in Boston and generated as previously described [26]. TRPC5^−/Y^ male and TRPC5^+/−^ female mice were bred to generate F_2_ mice with TRPC5^+/Y^, TRPC5^−/Y^, TRPC5^+/−^, and TRPC5^−/−^ genotypes. Homozygous mice that were screened and confirmed as TRPC5^+/Y^ (WT) and TRPC5^−/Y^ or ^−/−^ (TRPC5 KO) were used for further experimentation. Mice of both sexes were utilized in these studies. To determine the genotype and sex of animals, DNA was extracted from tissues of WT or TRPC5 KO 129S1/SvImJ mice. PCRs were prepared by mixing 150 ng of DNA and a set of primers (Table 2) into the AccuPower ProFi Taq PCR premix (BioNeer) and carried out in a C1000 Thermal Cycler (Bio-Rad) for 30 cycles (denaturation at 95 °C for 30 s, annealing at 59 °C for 30 s, and extension at 72 °C for 30 s). Amplified PCR products were separated on a 1.5% agarose gel and visualized on a Geldoc (Bio-Rad).

**Table 2:** List of primers for genotyping WT and *TRPC5* KO mice

Genes	Forward primer (5′ → 3′)	Reverse primer (5′ → 3′)
TRPC5 WT	GTAAGTGATACTAGGTATGGGGTATGGAGG	CACCAATCATGGATGTATTCCGTG
TRPC5 KO	GTAAGTGATACTAGGTATGGGGTATGGAGG	GTCGACACACGTATAAGGCATACTCTTG
SRY*	TTGTCTAGAGAGCATGGAGGGCCATGTCAA	CCACTCCTCTGTGACACTTTAGCCCTCCGA

*Sex-determining region Y

#### Animal Care and Seizure Induction

All animal experiments were approved by the Institutional Animal Care and Use Committee and followed a protocol approved by the Asan Medical Center. Adult male Sprague Dawley (SD) rats (8-week old, 240–270 g) were maintained under 12 h light/dark cycles. Seizures were induced by intraperitoneal injection of 10 mg/kg KA (Tocris) dissolved in saline. Animals were intraperitoneally injected with 100 μg/kg NU6027 or vehicle (10% DMSO in normal saline) 30 min after KA injection. Two hours after KA injection, seizure behavior was staged according to the classification system described by Zheng et al. [27]. After 2.5 h, seizures were stopped by an intraperitoneal injection of 50 mg/kg sodium phenytoin. Body weight and mortality were determined 24 h later.

#### Tissue Preparation and Cresyl Violet and Fluoro-Jade B Staining

Brains were harvested 24 h after KA injection and immediately frozen in dry ice. Coronal brain sections were cut using a cryostat microtome and fixed with 4% paraformaldehyde for 30 min. Neurons were stained with a 1% cresyl violet solution at room temperature for 10 min. To determine cell death, sections were immersed in 6% potassium permanganate for 5 min, followed by 30 min of incubation with 0.001% Fluoro-Jade B (FJB) solution (Histo-Chem Inc.). The numbers of cresyl violet and Fluoro-Jade B positive cells in the hippocampus, pyriform cortex, and thalamus were counted from both hemispheres in a total of 5 coronal sections, every 150 μm starting 2.8 mm from the bregma. Images were obtained using a fluorescent microscope (BX60; Olympus) equipped with a DP70 CCD camera (Olympus) at a wavelength of 480/525 nm (ex/em) under × 10 objective.

#### Experimental Design and Statistical Analysis

For all in vivo experiments, at least eight male animals were used. Two-tailed Student’s *t* test was used for statistical analysis. For all in vitro experiments, data analysis was performed using the Sigmaplot version 13.0 statistical package programs (SIGMASOFT). The number of replicates is three unless others state. Data is represented as mean ± SEM from three independent experiments performed in triplicate. Statistical analyses were performed using the unpaired 2-tailed Student’s *t* test for comparisons between two groups, and one-way ANOVA was used for comparisons of multiple groups. Data were considered significant at a *p* value of < 0.05. The degrees of freedom and *p* values are reported in the results section for each experiment.

## Results

### Cyclin-Dependent Kinase (CDK) Inhibitor, NU6027, Reduces Oxidative Stress-Induced Cell Death in Neurons

**Fig. 1:**
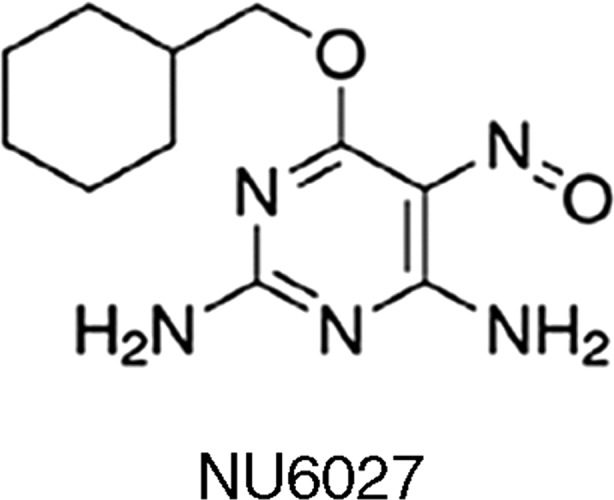
Chemical structure of NU6027

**Fig. 2:**
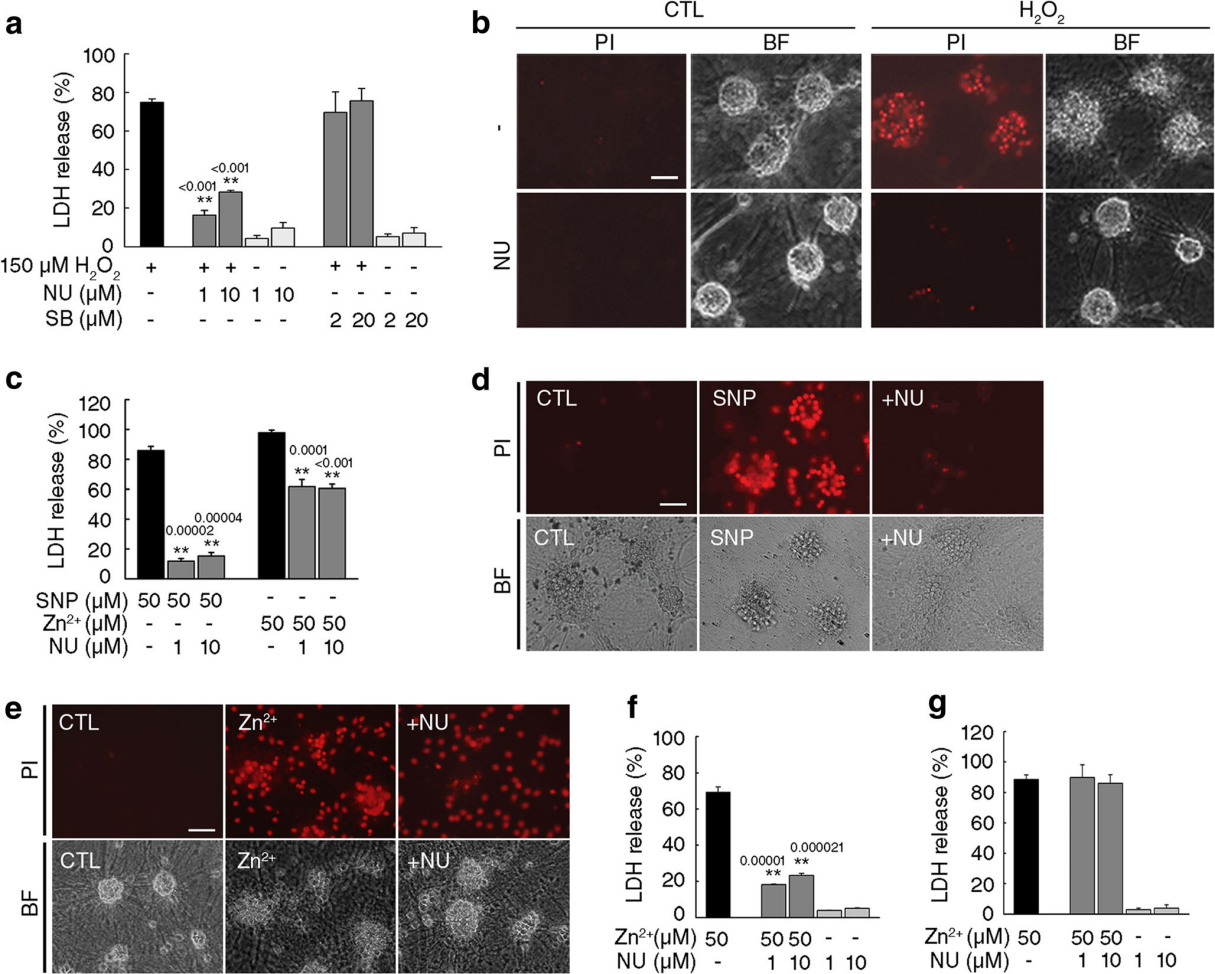
NU6027 reduces cell death induced by oxidative stresses in neurons but not in astrocytes. **a** Cell death in mixed cortical cultures containing neurons and astrocytes 24 h after treatment with 150 μM H_2_O_2_. The indicated concentrations of NU6027 (NU) or SB216763 (SB) were added 1 h before exposure to H_2_O_2_ (mean ± SEM, *n* = 3; ** indicates *p* < 0.001 compared with H_2_O_2_, 1-way ANOVA with Dunnet’s correction). **b** Representative images of bright field (BF) and propidium iodide (PI) staining of mixed cortical cultures exposed to 150 μM H_2_O_2_. Scale bar, 50 μm. **c** Cell death in mixed cortical cultures exposed to 50 μM sodium nitroprusside (SNP) or 50 μM ZnCl_2_ (Zn^2+^) for 24 h with or without the indicated concentrations of NU (mean ± SEM, *n* = 3; ** indicates *p* < 0.001 compared with Zn^2+^ or SNP, respectively, 1-way ANOVA with Dunnet’s correction). **d**, **e** Representative images of BF and PI staining of mixed cortical cultures exposed to 50 μM SNP (**d**) or 50 μM Zn^2+^ (**e**), in the presence or absence of 1 μM NU. Scale bar, 50 μm. **f**, **g** Cell death in pure neuronal cultures exposed to 50 μM Zn^2+^ (**f**), or in pure astrocyte cultures exposed to 50 μM Zn^2+^ (**g**) with or without NU (mean ± SEM, *n* = 3; ** indicates *p* < 0.001 compared with Zn^2+^,1-way ANOVA with Dunnet’s correction)

While searching for neuroprotective drugs effective against oxidative stress-induced cell death, we found that the CDK inhibitor NU6027 (Fig. 1a) markedly reduced H_2_O_2_-induced release of LDH, a quantitative biochemical marker for neuronal death, in primary mixed cortical cultures containing neurons and astrocytes (Fig. 2a). PI staining further confirmed that NU6027 markedly attenuated neuronal death induced by H_2_O_2_ (Fig. 2b). The protective effect of NU6027 seems to be CDK independent because several other CDK inhibitors, such as roscovitine, kenpaullone, olomoucine, and SU9516, were not effective in attenuating cell death induced by H_2_O_2_ (data not shown). Because many CDK inhibitors also block glycogen synthase kinase 3β (GSK3β) and GSK3β inhibitors protect against excitotoxicity in neurons [28, 29], we tested the GSK3β-specific inhibitor SB216763. However, SB216763 did not protect neurons from H_2_O_2_-induced cell death (Fig. 2a), suggesting that the protective effect of NU6027 was not due to the inhibition of GSK3β. In mixed cortical cultures, NU6027 virtually abolished oxidative neuronal cell death induced by the nitric oxide donor SNP, whereas only modest protection was observed against Zn^2+^-induced cell death (Fig. 2c). Only neurons were killed when mixed cultures were exposed to either H_2_O_2_ or SNP, which was almost completely blocked by NU6027 (Fig. 2b, d). In contrast, continuous exposure to Zn^2+^ killed both neurons and astrocytes, and protection by NU6027 was evident only in neurons (Fig. 2e). Additional experiments were performed to confirm whether NU6027 protects neurons but not astrocytes against oxidative injury. NU6027 efficiently blocked cell death induced by Zn^2+^ in pure neuronal cultures (Fig. 2f). However, it offered no protection in pure astrocyte cultures exposed to Zn^2+^ (Fig. 2g). These results indicate that the protective effect of NU6027 against diverse oxidative insults is highly selective to neurons.

### NU6027 Prevents Zinc-Dependent Elevation of Calcium by H_2_O_2_

**Fig. 3:**
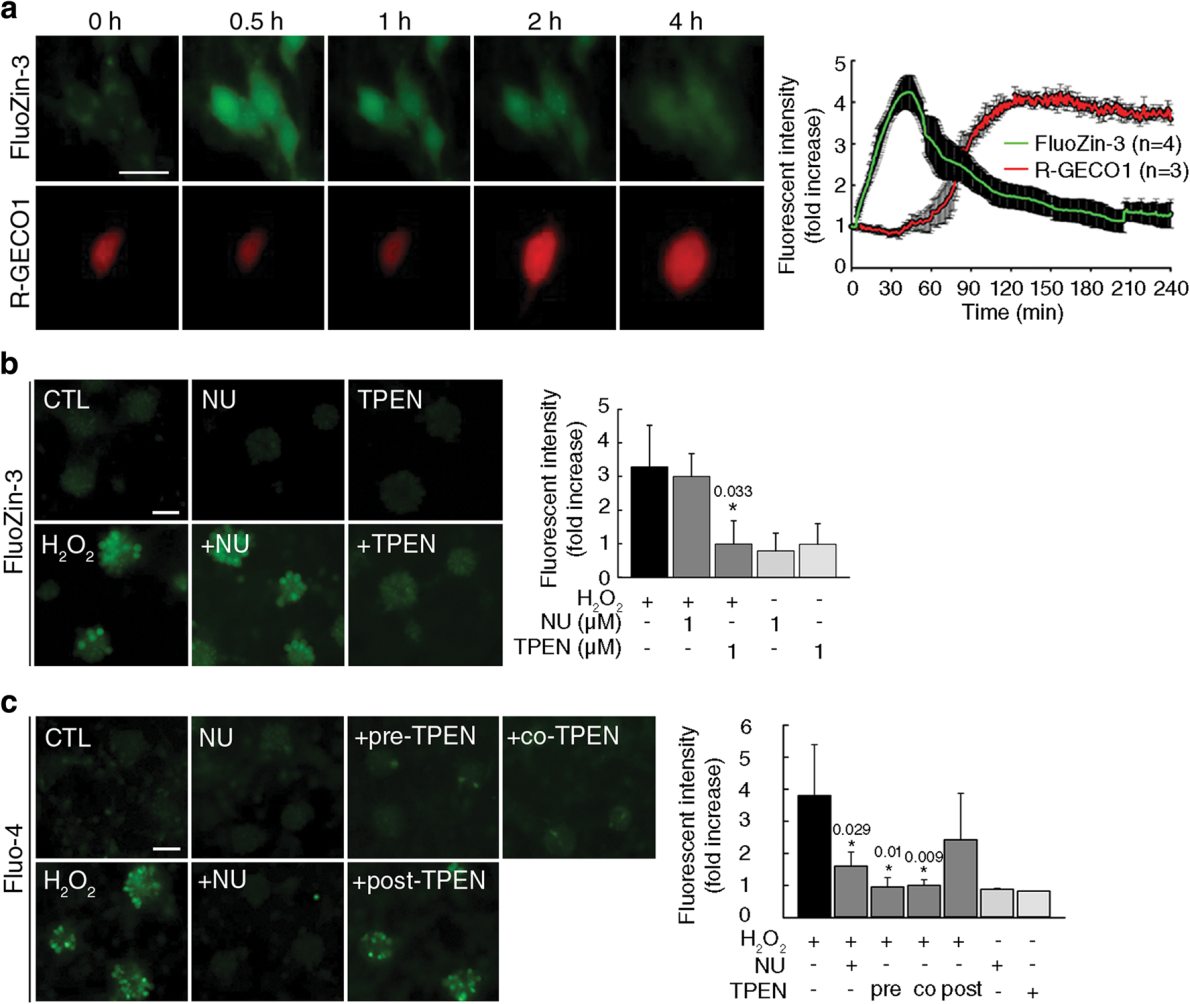
NU6027 blocks delayed Ca^2+^ influx induced by H_2_O_2_-triggered early increases in Zn^2+^. **a** Representative images (left) and quantification (right) of FluoZin-3 AM (FluoZin-3) and R-GECO1 signals from pure neuronal cultures treated with 25 μM H_2_O_2_. Scale bar, 20 μm. (mean ± SEM, *n* = 3 for FluoZin-3 AM, *n* = 4 for R-GECO1, 1-way ANOVA with Dunnet’s correction). **b** Mixed cortical cultures were pretreated with 1 μM NU or 1 μM *N*,*N*,*N*,*N*′-tetrakis (2-pyridylmethyl) ethylenediamine (TPEN) 1 h before exposure to 150 μM H_2_O_2_ for 30 min and then observed under a fluorescent microscope. Cells were stained with 2.5 μM FluoZin-3 for 30 min before observation. Scale bar, 50 μm. The bar graph (right) represents the normalized fluorescence intensity of FluoZin-3 (mean ± SEM, *n* = 3; * indicates *p* < 0.05 compared with H_2_O_2_, 1-way ANOVA with Turkey correction). **c** Mixed cortical cultures were pretreated with 1 μM NU 1 h before exposure to 150 μM H_2_O_2_ for 2 h (middle panel). The cells were exposed to 150 μM H_2_O_2_ for 2 h with 1 h pretreatment, cotreatment, or 1 h post-treatment with 1 μM TPEN (lower panel). Cells were stained with 2 μM Fluo-4 AM (Fluo-4) for 30 min prior to imaging. Scale bar, 50 μm. The bar graph (right) represents the normalized fluorescence intensity of Fluo-4 (mean ± SEM, *n* = 3; * indicates *p* < 0.05 compared with H_2_O_2_, 1-way ANOVA with Student–Newman–Keuls correction)

Increases in the intracellular concentrations of Zn^2+^ ([Zn^2+^]_*i*_) and Ca^2+^ [Ca^2+^]_*i*_ play key roles in oxidative cell death [11, 30, 31]. Therefore, we analyzed changes in the levels of these ions following H_2_O_2_ treatments in neurons loaded with FluoZin-3 AM, a Zn^2+^-specific fluorescent dye, or neurons transfected with p-CMV-RGECO1, a plasmid encoding a Ca^2+^-specific indicator peptide. Time-lapse imaging revealed that [Zn^2+^]_*i*_ began to increase almost immediately, reached maximal levels 30 min after H_2_O_2_ treatment, and gradually returned to basal levels over the following 3 h, whereas [Ca^2+^]_*i*_ started to increase 1 h after treatment and stayed at the maximum level for 2–4 h (Fig. 3a and Movie S1). Next, we examined the effect of NU6027 on H_2_O_2_-triggered increases in [Zn^2+^]_*i*_ and [Ca^2+^]_*i*_. Although TPEN, a membrane-permeable Zn^2+^ chelator, almost completely blocked the increase in [Zn^2+^]_*i*_, NU6027 did not block the increase in [Zn^2+^]_*i*_ when they were applied 1 h before H_2_O_2_ exposure (Fig. 3b). However, pretreatment with NU6027 substantially attenuated the elevation of [Ca^2+^]_*i*_ (Fig. 3c). Of note, pretreatment or cotreatment with TPEN significantly reduced the increase in [Ca^2+^]_*i*_ following H_2_O_2_ treatment. In contrast, 1 h post-treatment with TPEN following the peak of [Zn^2+^]_*i*_ had little effect on late increase in [Ca^2+^]_*i*_ (Fig. 3c). These results suggest that early increase in [Zn^2+^]_*i*_ is instrumental in inducing late increase in [Ca^2+^]_*i*_ during H_2_O_2_-induced cell death. Furthermore, NU6027 effectively inhibited increases in [Ca^2+^]_*i*_ but not in [Zn^2+^]_*i*_.

**Fig. 4:**
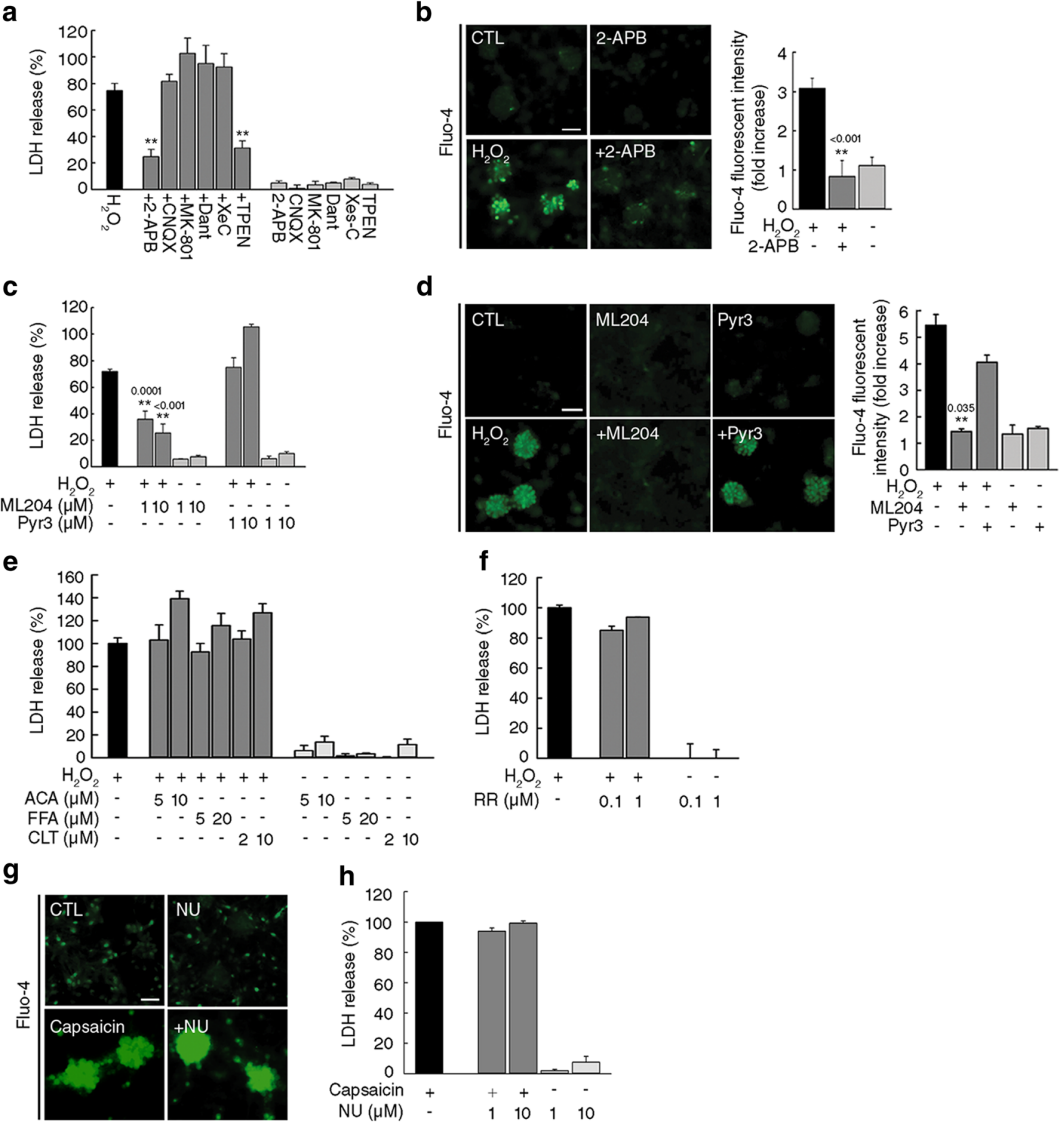
TRPC channels are responsible for H_2_O_2_-induced neuronal death. **a** Cell death in mixed cortical cultures exposed to 150 μM H_2_O_2_ for 24 h with or without 50 μM 2-aminoethyl diphenylborinate (2-APB), 10 μM 6-cyano-7-nitroquinoxaline-2,3-dione (CNQX), 10 μM MK-801, 10 μM dantrolene (Dant), 10 μM (−)-Xestosphongin C (Xes-C), or 1 μM TPEN (mean ± SEM, *n* = 3; ** indicates *p* < 0.001 compared with H_2_O_2_, 1-way ANOVA with Turkey correction). **b** Representative images (left) and quantification (right) of Fluo-4 signal from mixed cortical cultures exposed to H_2_O_2_ for 2 h with or without 2-APB. Scale bar, 50 μm. (mean ± SEM, *n* = 3; ** indicates *p* < 0.001 compared with H_2_O_2_, 1-way ANOVA with Dunnet’s correction). **c** Cell death in mixed cortical cultures exposed to H_2_O_2_ with or without ML204 or Pyr3 (mean ± SEM, *n* = 3; ** indicates *p* < 0.001 compared with H_2_O_2_, 1-way ANOVA with Dunnet’s correction). **d** Representative images (left) and quantification (right) of Fluo-4 signal from mixed cortical cultures exposed to H_2_O_2_ for 2 h with or without 1 μM ML204 or 1 μM Pyr3. Scale bar, 50 μm. (mean ± SEM, *n* = 4; ** indicates *p* < 0.001 compared with H_2_O_2_, 1-way ANOVA with Dunnet’s correction). **e**, **f** Cell death in mixed cortical cultures exposed to H_2_O_2_ with or without anthranilic acid (ACA), flufenamic acid (FFA), clotrimazole (CLT) (**e**), or ruthenium red (RR) (**f**) (mean ± SEM, *n* = 3). **g** Representative images of Fluo-4 signal in mixed cortical cultures exposed to 300 μM capsaicin for 3 h with or without NU. Scale bar, 50 μm. (mean ± SEM, *n* = 3). **h** Cell death in mixed cortical cultures exposed to 300 μM capsaicin for 24 h with or without NU (mean ± SEM, *n* = 3)

To determine the identity of receptors or ion channels that may mediate the Ca^2+^ influx following H_2_O_2_ exposure, we tested various antagonists, such as 2-APB (TRP channel blocker), CNQX (AMPA/KA receptor antagonist), MK-801 (NMDA receptor antagonist), dantrolene (ryanodine receptor antagonist), and XeC (IP3 receptor antagonist). Only 2-APB prevented H_2_O_2_-induced neuronal death and increase in [Ca^2+^]_*i*_ (Fig. 4a, b). Therefore, we tested the potential protective effects of inhibitors against TRPC, TRPM, and TRPV channels, which contribute to oxidative stress-induced neuronal death [9, 10, 32–34]. Treatment of neurons with ML204, a TRPC4 and TRPC5 blocker, attenuated H_2_O_2_-induced neuronal death and increase in [Ca^2+^]_*i*_, whereas Pyr3, a specific TRPC3 blocker, had no effect (Fig. 4c, d). Three TRPM inhibitors (anthranilic acid, flufenamic acid, and clotrimazole) and a TRPV inhibitor (ruthenium red) had no effect on H_2_O_2_-induced neuronal death (Fig. 4e, f). Moreover, NU6027 did not block the increase in [Ca^2+^]_*i*_ and neuronal death induced by capsaicin, a TRPV agonist (Fig. 4g, h). These data illustrate that among 2-APB-sensitive Ca^2+^-permeable channels, TRPC4 and TRPC5 are candidates for mediating the effects observed in this study.

### NU6027 Inhibits H_2_O_2_-Triggered Ca^2+^ Influx and Death by Antagonizing TRPC5

**Fig. 5:**
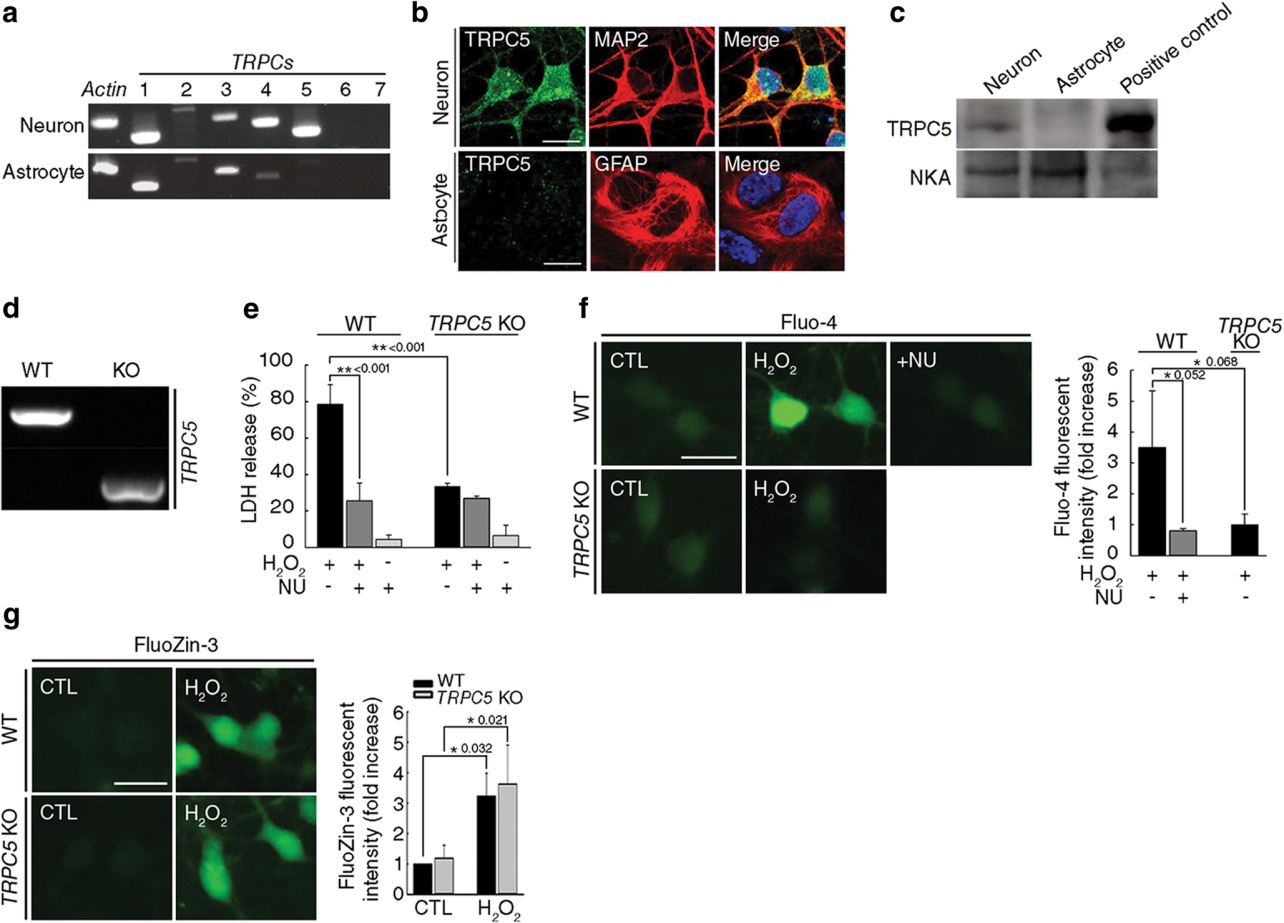
H_2_O_2_-triggered neuronal death and increases in [Ca^2+^]_i_ are diminished in TRPC5 KO mice. **a** *TRPC1-7* mRNA was analyzed by RT-PCR using specific primers in pure neuronal and astrocyte cultures. Actin was used as a housekeeping gene. **b** Immunofluorescence images of neurons and astrocytes labeled with TRPC5 antibodies. Microtubule-associated protein 2 (MAP2) is a neuronal marker and glial fibrillary acidic protein (GFAP) is an astrocytic marker. Scale bar, 20 μm. **c** The expression of TRPC5 protein was confirmed by Western blot analysis in membrane fractions purified from pure neuronal and astrocyte cultures. Membrane fractions extracted from mouse brain tissue were used as positive controls. Western blotting of Na(+)/K(+)-ATPase (NKA) confirms equal protein loading. **d** Genetic ablation of TRPC5 was determined by PCR analysis using specific primers. **e** Cell death in pure neuronal cultures from wild type (WT) and *TRPC5* knock-out (KO) littermate mice exposed to 40 μM H_2_O_2_ for 24 h with or without NU. (mean ± SEM, *n* = 3; ** indicates *p* < 0.001, 1-way ANOVA with Dunnet’s correction). **f**, **g** Pure neuronal cultures from WT and TRPC5 KO littermate mice were exposed to 40 μM H_2_O_2_ for 2 h or 30 min and stained with Fluo-4 (**f**) or FluoZin-3 (**g**), respectively. Representative images and quantification of fluorescence intensity for Fluo-4 or FluoZin-3 (mean ± SEM, *n* = 3; * indicates *p* < 0.05, 1-way ANOVA with Turkey correction)

**Fig. 6:**
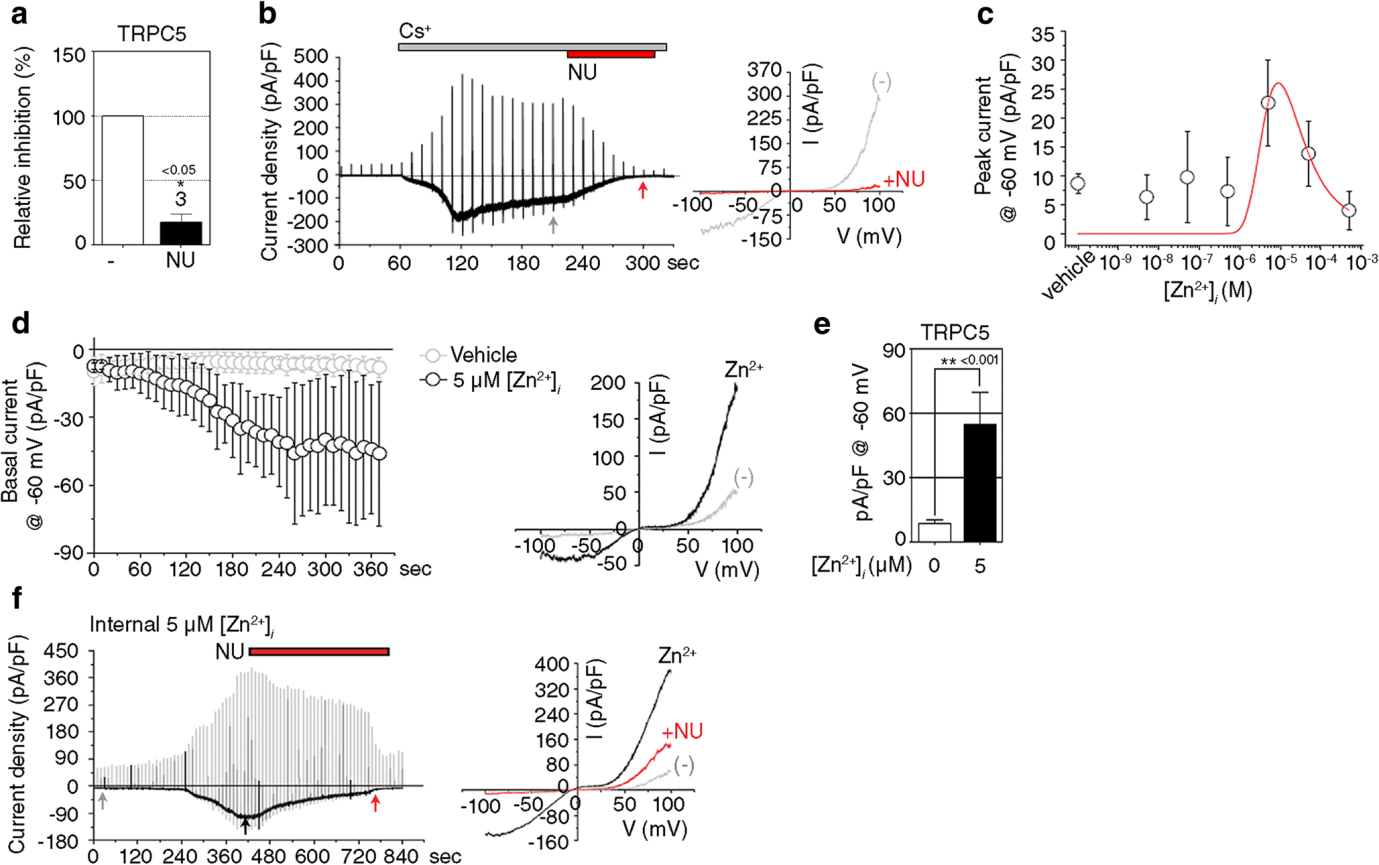
NU6027 inhibits TRPC5-mediated currents. **a** Inhibition of basal TRPC5 activity by treatment with 1 μM NU in HEK293 cells expressing TRPC5 (mean ± SEM, *n* = 3; * indicates *p* < 0.05 compared with control, 2-tailed t-test). **b** Representative current traces (left) and I–V curves (right) of basal TRPC5 activity and its inhibition by NU6027.**c** Dose-dependent changes in TRPC5 activity caused by the intracellular application of Zn^2+^ in TRPC5-expressing HEK293 cells (mean ± SEM, *n* = 4, 2-tailed t-test). **d** Quantitative changes in basal inward Na^+^ currents induced by intracellular 5 μM Zn^2+^ (mean ± SEM, vehicle, *n* = 8; Zn^2+^, *n* = 4, 2-tailed t-test). **e** Changes in basal inward Na^+^ currents induced by intracellular 5 μM Zn^2+^ (mean ± SEM, vehicle, *n* = 8; Zn^2+^, *n* = 4, ** indicates *p* < 0.001, 2-tailed t-test). **f** Representative current traces and I–V curves of intracellular Zn^2+^-induced TRPC5 activation and its inhibition by 1 μM NU6027

We next examined the expression of TRPC isoforms in cultures of pure neurons and pure astrocytes using RT-PCR. Interestingly, *TRPC5* mRNA was expressed exclusively in neurons and not in astrocytes (Fig. 5a). In accordance with this result, immunocytochemistry and Western blotting showed that TRPC5 proteins were expressed predominantly in neurons (Fig. 5b, c). To further elucidate the role of TRPC5 in H_2_O_2_-induced neuronal death and increase in [Ca^2+^]_*i*_, we used pure neuronal cultures from WT or TRPC5 KO mice (Fig. 5d). Neurons from TRPC5 KO mice were less sensitive to H_2_O_2_ toxicity than those from WT mice (Fig. 5e). Consistent with this result, exposure to H_2_O_2_ increased [Ca^2+^]_*i*_ in neurons from WT mice but not in TRPC5 KO neurons (Fig. 5f). In addition, treatment with NU6027 markedly attenuated H_2_O_2_-triggered neuronal death and increases in [Ca^2+^]_*i*_ in cultures from WT mice (Fig. 5e, f). *TRPC5* deficiency had no effect on H_2_O_2_-triggered increases in [Zn^2+^]_*i*_ (Fig. 5g), indicating that TRPC5 does not mediate increases in Zn^2+^ during oxidative stress. Thus, these data provide additional evidence that oxidative stress-sensitive TRPC5 mediates H_2_O_2_-triggered Ca^2+^ influx into neurons, which is inhibited by NU6027. To directly test this, we conducted electrophysiological recordings in HEK293 cells expressing mouse TRPC5. NU6027 almost completely blocked the increase in basal currents of TRPC5 induced by replacing external Na^+^ and K^+^ with Cs^+^, to which TRPC5 is highly permeable (Fig. 6a; *t*_(4)_ = 6.11861, *p* = 0.00181, paired *t* test, b). To determine the effect of intracellular Zn^2+^ on TRPC5 activity, we infused cells with Zn^2+^ at varying concentrations. Based on the curve, the half-maximal increase in TRPC5 current occurred at approximately 5 μM Zn^2+^ (Fig. 6c). Next, 5 μM Zn^2+^ was infused intracellularly, which increased the basal Na^+^ inward currents of TRPC5 by approximately 5-fold compared with currents obtained using the Zn^2+^-free pipette solution (Fig. 6d, e; *t*_(10)_ = 4.51, *p* = 0.00112, paired *t* test). The peak TRPC5 current induced by intracellular Zn^2+^ was attenuated by NU6027 (Fig. 6f). These results support the possibility that intracellular Zn^2+^ contributes to the gating of TRPC5, which is directly blocked by NU6027.

### NU6027 Ameliorates Cell Death Induced by Kainate in Rat Seizure Model

**Fig. 7:**
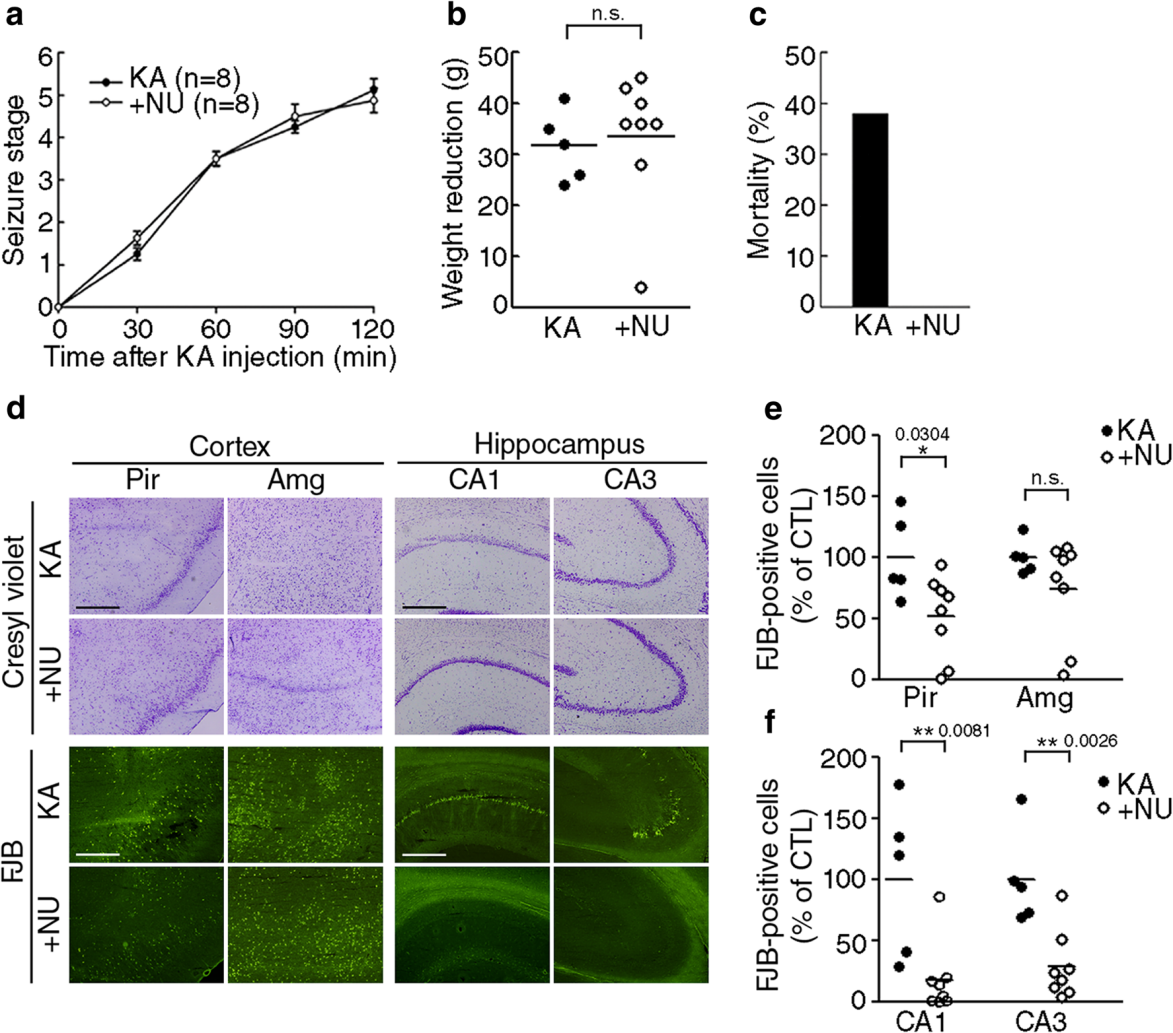
NU6027 reduces cell death in rat brain after kainic acid-induced seizure. **a** Seizure stages were evaluated in SD rats injected with 10 mg/kg KA for 2 h (mean ± SEM, *n* = 8). Vehicle or 100 μg/kg NU was injected 30 min after kainic acid (KA) exposure. **b**, **c** Body weight (**b**) and mortality (**c**) were measured 24 h after seizure induction (mean ± SEM, KA, *n* = 5; NU, *n* = 8, 2-tailed t-test). **d** Live cells were stained with 1% cresyl violet, and dead cells were visualized by staining with 0.001% FJB. **e**, **f** The bar graphs represent the number of FJB-positive cells in the piriform(Pir) cortex and amygdala (Amg, **e**) and the hippocampal CA1 and CA3 regions (**f**) (mean ± SEM, KA, *n* = 5; NU, *n* = 8, * indicates *p* < 0.05 and ** indicates *p* < 0.001 compared with KA, 2-tailed t-test)

Finally, we examined the protective effect of NU6027 in a rat model of KA-induced prolonged seizures. NU6027 altered neither seizure severity nor body weight reductions in surviving rats compared with vehicle-injected animals (Fig. 7a, b; *t*_(11)_ = 0.2983, *p* = 0.7710, unpaired *t* test). However, mortality was markedly reduced in NU6027-treated rat, from 38 to 0% (Fig. 7c). In addition, live cell staining with cresyl violet revealed that NU6027 treatment substantially reduced kainate seizure-induced neuronal loss in the pyriform cortex, amygdala, and hippocampus (Fig. 7d). This was supported by the significant reduction in the number of FJB-positive dead neurons following NU6027 treatment (Fig. 7e; *Pir t*_(11)_ = 2.484, *p* = 0.0304, *Amg t*_(11)_ = 1.364, *p* = 0.2000, unpaired *t* test, f; CA1 *t*_(11)_ = 3.224, *p* = 0.0081, CA3 *t*_(11)_ = 3.877, *p* = 0.0026, unpaired *t* test).

## Discussion

The main findings of this study are that neuronal death induced by oxidative stress, such as H_2_O_2_, is mediated by early transient increases in Zn^2+^ and delayed prolonged increases in Ca^2+^ and that these events are mechanistically linked. The increase in free Zn^2+^ levels appears to be necessary for the delayed accumulation of Ca^2+^ because the addition of a Zn^2+^-specific chelator, TPEN, while Zn^2+^ levels are increasing, completely blocks the Ca^2+^ influx. Hence, it is likely that delayed and prolonged Ca^2+^ influx is the final effector of cell death in cases of oxidative neuronal injury, whereas transient Zn^2+^ increases are critical for switching cells to Ca^2+^ entry.

Several lines of evidences from our results suggest that the delayed and prolonged influx of Ca^2+^ into cultured cortical neurons is mediated by TRPC5. First, among tested inhibitors of intracellular Ca^2+^ increases, only 2-APB and ML204 were effective in curtailing Ca^2+^ increases as well as cell death following H_2_O_2_ exposure. Although 2-APB is a broad-spectrum inhibitor of TRP class channels, ML204 is a specific inhibitor of TRPC4 and TRPC5 channels. Furthermore, neither inhibitors of TRPM channel, anthranilic acid, flufenamic acid, and clotrimazole, nor an inhibitor of TRPV, ruthenium red, showed any effect at all, making it unlikely that these TRP channels are involved. Second, NU6027, a CDK inhibitor, which blocked the delayed increase in Ca^2+^ and neuronal death induced by H_2_O_2_, directly reduced TRPC5-mediated channel currents in the channel-transfected HEK293 cells. It is unlikely that CDK inhibition is the underlying mechanism for this effect because other more potent CDK inhibitors did not have similar effects. Third, TRPC5 was selectively expressed in neurons but not in astrocytes in primary cortical cultures, which is consistent with the selective protection of zinc-exposed neurons by NU6027. Finally, knockout of TRPC5 was sufficient to reduce the delayed Ca^2+^ influx and cell death induced by H_2_O_2_ in cortical neurons.

TRPC channels belong to a family of TRP channels. TRP channels have many cysteine and histidine residues of which modifications activate TRP channels. For example, nitrosylation of Cys553/Cys558 residues in pore-forming region activates TRPC5, TRPV1, TRPV3, and TRPV4 [35]. Glutathionylation of intracellular N-terminal Cys176/Cys178 activates TRPC5 [32]. Another notable feature of TRP channels is that they can be regulated by metal ions, including Zn^2+^ [36]. Intracellular Zn^2+^ activates TRPA1 by modulating specific intracellular Cys641, Cys1021, and His983 [37]. It was also reported that extracellular Zn^2+^ activates TRPV1, though precise sites of modification are not elucidated in this report [38]. Although detailed information regarding the gating mechanism for TRPC5 is not yet available, our results suggest the intriguing possibility that increase in intracellular Zn^2+^ contributes to activation of TRPC5. It should be examined in the future whether Zn^2+^ directly participates in the gating of TRPC5, as occurs in that of TRPA1 in which the binding of Zn^2+^ to cytosolic cysteine and histidine residues is responsible for gating.

Mechanisms involved in oxidative neuronal cell death have been investigated intensively over the last several decades. Although Ca^2+^ overload is a major ionic mechanism mediating cell death, Zn^2+^ dyshomeostasis has been proposed as an additional mechanism later. During oxidative stress, Zn^2+^ binding proteins, most notably metallothioneins, can release Zn^2+^ [39, 40]. Prolonged Zn^2+^ dyshomeostasis can activate cellular processes, such as mitochondrial damage, NADPH oxidase and nitric oxide synthase activation, PARP activation, and lysosomal membrane permeabilization, which eventually lead to cell death [41–43]. In the present study, we observed that even transient increases in free Zn^2+^ levels were a prerequisite for delayed and prolonged Ca^2+^ influx, as discussed above. Hence, even in cases where Zn^2+^ dyshomeostasis is not severe enough to cause cell death by itself, Zn^2+^ may still play a large role in oxidative neuronal cell death by permitting a large Ca^2+^ influx though TRPC channels.

TRPC channels are subdivided into two groups based on sequence homology and functional properties. One is TRPC1/4/5 and the other is TRPC3/6/7 of which homo- or hetero-tetramer can be regulated by receptor stimulation [44]. Homomeric TRPC5 channel has been implicated in pathological roles for seizure and excitotoxicity [45]. In TRPC5 KO mice, seizure and neuronal cell death induced by pilocarpine was significantly reduced [34]. In cortical lesions of the focal cortical dysplasia, common intractable epilepsy in both pediatric and adult patients, the expression of TRPC5 is significantly increased in glutamatergic and GABAergic neurons [46]. Consistently, in our results, *TRPC5* mRNA and protein were predominantly expressed in neurons than astrocytes, cell death was dependent to the expression of TRPC5, and NU6027, an inhibitor of TRPC5, reduced neuronal death in cortical cultures. Our results also showed that NU6027 decreased cell death and mortality in a kainate model of epileptic brain damage. TRPC5 plays pathophysiological roles in other diseases, for example pain and anxiety, diabetic nephropathy, cardiovascular disease, rheumatoid arthritis, and cancer [26, 47–50]. The pharmacological tools available to unveil its pathophysiological activities are limited. Small-molecular inhibitors, such as SKF-93635 and 2-APB, nonspecifically inhibit all TRPC channels and other ion channels [51]. ML204 and the anti-histamine clemizole hydrochloride have a higher selectivity for TRPC4 than TRPC5 and inhibit channels at micromolar concentrations [52]. Pico145, a recently reported TRPC1/4/5-specific inhibitor with picomolar range of potency, has not been verified its inhibitory activity in in vivo systems [53]. Therefore, there is a pressing need for potent and specific inhibitory tool compounds. Our results suggest that NU6027 is a useful template in designing an effective inhibitor of TRPC5.

In conclusion, our results demonstrate that oxidative stress-induced neuronal cell death involves Zn^2+^-triggered delayed Ca^2+^ increases in neurons through TRPC5. NU6027 may directly block TRPC5-mediated Ca^2+^ influx in a CDK-independent manner. The time course of increase in Ca^2+^ suggests that inhibitors of TRPC5 have neuroprotective effects even when administered at later stages of acute neuronal injuries, such as epilepsy.

## Electronic Supplementary Material

ESM 1 (AVI 5506 kb)
